# Spectrum of glucose-6-phosphate dehydrogenase (G6PD) mutations and trends in hemoglobin levels among adult dengue patients in Thailand

**DOI:** 10.1371/journal.pone.0332039

**Published:** 2025-09-18

**Authors:** Supat Chamnanchanunt, Beatriz Aira C. Jacob, Vipa Thanachartwet, Varunee Desakorn, Natsamon Singha-art, Duangjai Sahassananda, Kamonwan Chamchoy, Naveen Eugene Louis, Muawiaa Ahmed Hamza, Nurriza Ab Latif, Syazwani Itri Amran, Henry A. F. Stephens, Wang Nguitragool, Usa Boonyuen

**Affiliations:** 1 Department of Clinical Tropical Medicine, Faculty of Tropical Medicine, Mahidol University, Bangkok, Thailand; 2 Department of Molecular Tropical Medicine and Genetics, Faculty of Tropical Medicine, Mahidol University, Bangkok, Thailand; 3 Information Technology Unit, Faculty of Tropical Medicine, Mahidol University, Bangkok, Thailand; 4 Princess Srisavangavadhana Faculty of Medicine, Chulabhorn Royal Academy, Bangkok, Thailand; 5 Department of Biosciences, Faculty of Science, Universiti Teknologi Malaysia (UTM), Johor Bahru, Malaysia; 6 Faculty of Medicine, King Fahad Medical City, Riyadh, Saudi Arabia; 7 Department of Renal Medicine, University College London (UCL), Royal Free Hospital, London, United Kingdom; Menzies School of Health Research, AUSTRALIA

## Abstract

Glucose-6-phosphate dehydrogenase (G6PD) deficiency is the most common enzymopathy in humans that may exacerbate clinical outcomes during viral infections such as dengue, particularly in regions where both conditions are endemic. This study aimed to characterize the spectrum of *G6PD* mutations and explore trends in hemoglobin levels among adult dengue patients in Thailand. Samples from 231 adult patients diagnosed with dengue were analyzed. G6PD deficiency was identified in 24 individuals (10.4%), while *G6PD* mutations were detected in 111 patients (48.1%). The most frequently observed mutations include a combination of synonymous and intronic mutations (c.1311C > T and c.1365-13T > C), compound mutation of *G6PD* Viangchan (c. 871G > A, c.1311C > T and c.1365-13T > C), and a deletion variant (c.486−34delT). Additionally, a novel variant, c.1439T > C, was identified and named “*G6PD* Phaya Thai”. Patients carrying *G6PD* mutations exhibited different hemoglobin level trends compared to those without mutations. Specifically, while hemoglobin levels increased from the febrile to critical phase in patients without mutations, a significant decline was observed in mutation carriers. Median hemoglobin levels differed significantly between the two groups during both the febrile and critical phases (*p* = 0.02 and *p* < 0.001, respectively). Biochemical and structural analyses of uncharacterized variants, G6PD Phaya Thai and G6PD Viangchan+Chinese-5, suggested structural instability as a possible mechanism for the observed deficiency. These findings highlight the need for further investigation into the potential role of *G6PD* variants in dengue-related anemia. Routine G6PD screening and continuous hemoglobin monitoring may help identify individuals at risk of hemoglobin decline and guide supportive care strategies in dengue-endemic regions.

## Introduction

Dengue is a major public health concern in tropical and subtropical regions, with global incidence rising from 26.45 million to 58.96 million cases and related deaths increasing from 14,315–29,075 between 1990 and 2021 [1]. It is caused by the dengue virus (DENV-1, DENV-2, DENV-3, DENV-4), an RNA virus belonging to the *Flaviviridae* family [2]. In Thailand, dengue outbreaks result in 40,000 to over 100,000 reported cases, with a mortality rate of 0.06‒0.13% over the past decade [3]. While most dengue infections are asymptomatic or mild, a small percentage of patients (less than 5%) develop severe complications such as dengue hemorrhagic fever (DHF) or dengue shock syndrome (DSS) [4,5]. The risk of developing DHF appears to increase with sequential infections involving specific serotypes and the time interval between primary and secondary infections [6–8]. However, since only a relatively small fraction of dengue infections progress to DHF or DSS, host factors are believed to play crucial roles in dengue pathogenesis [9]. Genetic polymorphisms in several genes have been found to confer increased susceptibility to severe manifestations. These include genes encoding leukocyte antigen class I [10,11], mannose binding lectin 2 [12,13], interferon gamma [13,14], major histocompatibility complex class I polypeptide-related sequence B, and phospholipase C epsilon 1 [13,15,16]. Another host factor of interest is glucose-6-phosphate dehydrogenase (G6PD) deficiency, a common X-linked enzymopathy that may influence immune responses and redox homeostasis during infection [17,18].

G6PD deficiency is the most common human enzymopathy, affecting approximately 500 million people worldwide [19]. G6PD plays a crucial role in the cellular antioxidant defense system by producing reduced nicotinamide adenine dinucleotide phosphate (NADPH), a coenzyme essential for maintaining reduced glutathione levels [20]. Mutations in the *G6PD* gene can lead to decreased enzyme activity and structural instability, limiting NADPH formation and consequently impairing the cell’s ability to counteract oxidative stress [21]. This is especially relevant during infections, where reactive nitrogen and oxygen species are essential for pathogen clearance. In the context of dengue, oxidative stress and altered redox balance in G6PD-deficient cells may promote viral replication and impair immune cell function, making them more vulnerable [22–24]. Previous studies have reported increased susceptibility to DENV-2 infection and altered granulocyte function in G6PD-deficient individuals [17,18]. Furthermore, a higher prevalence of G6PD deficiency was observed among male DHF patients in Bangkok compared to the general population, although no direct association with disease severity was established [25]. Similarly, a study in Myanmar children found no statistically significant correlation between G6PD deficiency or specific genotypes‒particularly the *G6PD* Mahidol variant‒and the severity of dengue infection [26].

In addition to potential immune implications, G6PD deficiency may increase the risk of hemolysis during dengue infection, especially during the critical phase when inflammatory responses peak [27]. Hemoglobin levels often fluctuate across the febrile, critical, and recovery phases of dengue. Initially, hemoconcentration from plasma leakage may mask underlying hemolysis, while later phases can involve vascular permeability and immune-mediated destruction of red blood cells, potentially causing a rapid drop in hemoglobin levels [25,28]. This can increase the risk of anemia and related complications, particularly in individuals with predisposing conditions such as G6PD deficiency.

In Thailand, G6PD deficiency affects an estimated 3‒18% of the population, with significant ethnic and regional variability and over 20 known mutations [29–33]. Despite the overlap between dengue and G6PD deficiency, relatively few studies have investigated the genetic spectrum of *G6PD* variants in Thai dengue patients or explored trends in hemoglobin levels in relation to genotype.

This study aimed to characterize the spectrum of *G6PD* mutations in adult dengue patients in Thailand and to explore differences in hemoglobin level dynamics across dengue phases in individuals with and without *G6PD* mutations. A novel variant, *G6PD* Phaya Thai (c.1439T > C), and a compound mutation, *G6PD* Viangchan+Chinese-5, were identified and subjected to biochemical and structural characterization to assess their potential functional impact. These findings contribute to our understanding of *G6PD* genetic diversity in a dengue-endemic population and offer insight into possible hematologic implications during infection.

## Methods

### Ethics statement and sample collection

Samples were originally collected from dengue patients between 2 May 2013 and 30 December 2014 under a research protocol approved by the Human Ethics Committee of the Faculty of Tropical Medicine, Mahidol University (MUTM 2013-007-11). Written informed consent was obtained from all participants at the time of collection, including permission for future use of their specimens in research.

The study included adult patients (≥18 years) seeking treatment for dengue at the Hospital for Tropical Diseases, Bangkok, Thailand, who met the following criteria: fever for less than seven days and at least two of the following symptoms: headache, retro-orbital pain, muscle or joint pain, rash, bleeding, low white blood cell count, low platelet count, or rising red blood cell volume‒along with a positive dengue test (dengue virus nonstructural protein test, immunoglobulin M test, or real-time reverse transcriptase-polymerase chain reaction; RT-PCR) [34]. A total of 231 subjects (123 males and 108 females) were included. Blood samples were obtained via venipuncture into EDTA tubes from patients who met all inclusion criteria, then aliquoted and stored at −80 °C until further analysis. The median time of sample collection was 4 days after symptom onset (interquartile range (IQR): 3–5 days).

The stored samples were later retrieved and subjected to genetic analysis between 20 November and 24 December 2022 under a follow-up protocol (MUTM 2021-075-02), which received separate ethical approval from the same committee. This subsequent protocol was designed to extend the original study objectives by incorporating advanced molecular analyses. All data were fully anonymized, and the authors had no access to any information that could identify individual participants.

### Dengue phase and hemoglobin detection

Dengue disease progression was monitored by classifying time points based on the initial fever onset. The febrile phase was defined by the presence of high-grade fever accompanied by one or more of the following symptoms: headache, retro-orbital pain, myalgia, or arthralgia. The critical phase was identified based on clinical, hematological, or radiological findings that warranted close patient monitoring. These include the appearance of warning signs suggestive of plasma leakage, a progressive rise in hematocrit of at least 20% from baseline, a platelet count dropping below 100 x 10⁹/µL, or radiological evidence of fluid leakage detected by chest x-ray or ultrasound. The recovery phase began with the resolution of acute symptoms and the onset of clinical improvement.

Hemoglobin levels were analyzed in relation to specific time points of illness. Complete blood count tests were performed using an automated hematology analyzer (Mindray® BC-6200), which was calibrated daily before testing.

### Phenotypic screening of G6PD deficiency

Blood samples were used to test for G6PD deficiency. The prevalence of G6PD deficiency was determined by measuring enzyme activity using the water-soluble tetrazolium salts (WST-8) assay, according to a previous study [35]. In brief, 2 µL of blood was mixed with a reaction buffer containing 20 mM Tris-HCl (pH 8.0), 10 mM MgCl_2_, 500 μM glucose-6-phosphate (G6P), 100 μM NADP^+^, and 100 μM WST-8 (Sigma-Aldrich, Darmstadt, Germany). The reaction was monitored by measuring absorbance at 450 nm using a microplate reader (Sunrise; Tecan, Männedorf, Switzerland). Hemoglobin concentration was determined using Drabkin’s reagent (Sigma-Aldrich), following the manufacturer’s instructions. The activity of G6PD was expressed as units per gram hemoglobin (U/gHb). The adjusted male median (AMM) was calculated by first determining the median G6PD activity among all male participants, then excluding those with activity less than or equal to 10% of the median. The resulting AMM was defined as 100% enzyme activity and used as the reference for classification [36].

### *G6PD* genotyping

The genomic DNA was extracted using a QIAamp DNA Blood Mini Kit (QIAGEN, Hilden, Germany) in accordance with the manufacturer’s instructions. Following previously described protocols [37], *G6PD* genotyping was carried out on all samples using high-resolution melting curve (HRM) assays to detect 15 mutations that are common in Asian populations, namely, *G6PD* Gaohe (c.95A > G), *G6PD* Aures (c.143T > C), *G6PD* Songklanagarind (c.196T > A), *G6PD* Chinese-4 (c.392G > T), *G6PD* Valladolid (c.406C > T), *G6PD* Mahidol (c.487G > A), *G6PD* Mediterranean (c.563C > T), *G6PD* Coimbra (c.592C > T), *G6PD* Viangchan (c.871G > A), *G6PD* Chinese-5 (c.1024C > T), *G6PD* Union (c.1360C > T), *G6PD* Canton (c.1376G > T), intron 5 deletion (c.486−34delT), synonymous mutation (c.1311C > T) and intron 11 mutation (c.1365-13T > C).

Samples with G6PD activity <80% of the AMM but no mutations detected by HRM assays were amplified for the *G6PD* gene and sent for sequencing (1st BASE; Apical Scientific, Selangor, Malaysia). PCR amplification was carried out following a previous report [38].

### Biochemical and structural characterization of G6PD variants

To understand the molecular mechanisms underlying enzyme deficiency in the uncharacterized double missense mutation G6PD Viangchan+Chinese-5 and the single missense mutation c.1439T > C (designated as G6PD Phaya Thai), these two variants were created and thoroughly characterized in terms of biochemical and structural properties.

#### Site-directed mutagenesis and protein expression and purification.

G6PD variants were created by site-directed mutagenesis with the pET28a-G6PD wild-type (WT) as a template. Primers used for site-directed mutagenesis are listed in S1 Table in S1 File. The PCR conditions for site-directed mutagenesis were previously described [37]. The presence of desired mutations was verified by DNA sequencing.

G6PD protein was produced in *E. coli* BL21 (DE3) and purified to homogeneity using immobilized metal affinity chromatography, as described before [37].

#### Determination of steady-state kinetic parameters of G6PD variants.

Steady-state kinetic parameters of G6PD variants were determined to assess the effects of mutations on substrate affinity and catalytic activity by monitoring the formation of NADPH at 340 nm using a spectrophotometer. To determine the *K*_m_ for glucose-6-phosphate (G6P), the concentration of NADP^+^ was fixed at 100 μM while varying concentrations of G6P from 2.5 to 1,000 µM and to determine the *K*_m_ for NADP^+^, the concentration of G6P was fixed at 500 μM while varying concentrations of NADP^+^ from 1 to 200 µM.

#### Determination of secondary structure of G6PD variants.

The effect of mutations on secondary structure of G6PD proteins was analyzed using circular dichroism (CD). Far UV-CD spectra of the G6PD variants (0.1 mg/mL) were recorded in a 1 mm path-length quartz cuvette at 25 °C using a Jasco spectrometer, model J-815, equipped with a Peltier temperature control system.

#### Structural stability analyses of G6PD variants.

Structural stability analyses of G6PD variants were carried out to determine how mutations affect G6PD structural stability. X-ray crystallography revealed that the human G6PD protein has two NADP^+^-binding sites, the second of which is known as the NADP^+^-structural site, which plays a key role in structural stabilization and is located right next to the dimer interface [39]. Therefore, structural stability analyses of G6PD variants were examined under different NADP^+^ concentrations (0, 10 and 100 µM).

Thermal unfolding methods were used to assess the impact of different mutations on protein structure, stability and activity. Firstly, protein stability was monitored upon increasing temperatures. The assay was performed in a 20 μL reaction, containing protein at a concentration of 0.25 mg/ml and 5 × SYPRO Orange Protein Gel Stain (Thermo Fisher Scientific, San Jose, CA, USA). The reaction mixtures were subjected to increasing temperatures ranging from 20 to 80 °C in a LightCycler 480 real-time PCR machine (Roche, Mannheim, Germany) in the presence of various concentrations of NADP^+^ (0, 10 and 100 µM). The melting temperature (*T*_*m*_) of each G6PD variant was calculated and defined as the temperature at which half of the protein unfolded.

Secondly, thermal inactivation assay was carried out where the enzyme was incubated for 20 min at temperatures ranging from 25 to 65 °C in a Thermocycler (Eppendorf, Hamburg, Germany) in the presence of various concentrations of NADP^+^ (0, 10 and 100 µM). The residual enzyme activity was measured and expressed as a percentage of the activity of the same enzyme incubated at 25 °C. T_1/2_ was defined as the temperature at which the enzyme loses 50% of its activity.

Structural stability of G6PD variants upon chemical denaturation was also determined. Guanidine hydrochloride (Gdn-HCl) is a chaotropic molecule that causes protein denaturation by disrupting the noncovalent interactions of the proteins and thereby altering tertiary structure. Protein unfolding upon exposure to increasing Gdn-HCl concentrations can be used to assess structural stability. The protein was treated with different concentrations of Gdn-HCl (0 to 0.5 M) in the presence of various concentrations of NADP^+^ (0, 10 and 100 µM) at 37 °C for 2 h. The residual enzyme activity was measured and expressed as a percentage of the activity of the same enzyme incubated without Gdn-HCl. C_1/2_ was defined as the concentration of Gdn-HCl at which the enzyme loses 50% of its activity.

Finally, proteolytic susceptibility assay was used to assess the unfolding/misfolding effect of G6PD mutations on the protein structure. The protein was treated with trypsin (0.5 mg/mL) for 5 min at 25 °C in the presence of various concentrations of NADP^+^ (0, 10 and 100 µM). The residual enzyme activity was measured and expressed as a percentage of the activity of the same enzyme incubated without trypsin.

### Molecular docking and molecular dynamic simulations

G6PD dimeric structure in complex with G6P and NADP^+^ ligands was constructed using molecular docking approach with AutoDock 4.2 software [40]. *In silico* site-directed mutagenesis was performed to create G6PD mutants using PyMOL software (PyMOL Molecular Graphics System, Version 3.0 Schrödinger, LLC.). Both WT and mutant enzyme structures were prepared using pdb2gmx utility and the GROMOS96 54a7 force field while the ligand topology files were prepared by using the Automated Topology Builder [41,42]. All simulations were performed using the GROMACS 2018.1 package, as reported in a previous G6PD structural study [43]. Post-simulation structural analysis of critical regions, including mutation sites, dimer and tetramer interfaces, and protein–ligand affinities, was performed using various trajectory analyses such as root mean square deviation (RMSD), root mean square fluctuation (RMSF), hydrogen bond analysis, radius of gyration (Rg), and solvent accessible surface area (SASA). Visual inspection and graphical presentations of the systems were performed using Discovery Studio Visualizer v21.1.0.20298.

### Statistical analysis

The data were statistically analyzed using SPSS program version 18.0 and GraphPad Prism. Descriptive analysis was presented as number (n) and frequencies (%). Hemoglobin levels were expressed as median and IQR. Pairwise comparison of continuous variables was performed using the Mann‒Whitney U test and Wilcoxon Signed rank test for two independent and dependent samples, respectively. A *p*-value < 0.05 was considered statistically significant.

## Results

### Demographic characteristics, G6PD activity and genotypes in dengue patients

A total of 231 patients with confirmed dengue infection were enrolled in the study (Table 1). The median age was 24 years (IQR: 19–36), with a slight predominance of males (53.2%). Of all participants, 17.3% reported a previous history of dengue infection and 32.2% had at least one chronic illness. Self-medication prior to hospital admission was reported by 90.0% of patients, with acetaminophen being the most common used drug, followed by NSAIDs and antibiotics. The median time of dengue testing was 4 days after symptom onset (IQR: 3‒5). Diagnosis was established by NS1 antigen detection in 79.2%, IgM serology in 29.4%, and RT-PCR in 3.0%. The most common reported symptoms were myalgia (88.3%), headache (74.9%), and fatigue (61.9%). On admission, the median body temperature was 38.0 °C (IQR: 37.4–38.7). Hepatomegaly was identified in 62.3% of cases, while jaundice and rash were observed in 1.7% and 7.4% of patients, respectively. The median duration of hospitalization was 4 days (IQR: 2‒5), typically covering the febrile to the critical phase. The febrile phase, usually occurring on days 2–5 of illness, was managed with supportive care. Oral fluid intake was encouraged, and intravenous fluids were administered when necessary with careful monitoring to prevent volume overload. The critical phase, which generally begins around the time of defervescence (typically days 3–7), lasts 24–48 hours. During this period, close monitoring and careful fluid management using isotonic crystalloid solutions were essential.

**Table 1:** Baseline characteristics among patients with dengue infection.

Characteristics	All (n = 231)		G6PD genotype				*p*-value
			Mutation detected (n = 111)		No mutation detected (n = 120)		
Age	24	(19‒37)	23	(19‒35 )	25	(20–37)	0.138
Living area: Bangkok	181	(78.4%)	90	(81.1%)	91	(75.8%)	0.343
Travel history	71	(30.7%)	33	(29.7%)	38	(31.7%)	0.777
Previous dengue infection	40	(17.3%)	25	(22.5%)	15	(12.5%)	0.054
Preexisting diseases	74	(32.0%)	34	(30.6%)	40	(33.3%)	0.675
Self-medication							
Acetaminophen	208	(90.0%)	102	(91.9%)	106	(88.3%)	0.389
NSAIDs	18	(7.8%)	9	(8.1%)	9	(7.5%)	0.528
Antibiotics	18	(7.8%)	7	(6.3%)	11	(9.2%)	0.469
PPIs	11	(4.8%)	8	(7.2%)	3	(2.5%)	0.124
Norethisterone	1	(0.4%)	0	–	1	(0.8%)	0.519
Dengue diagnostic approaches							
NS1 antigen detection	183	(79.2%)	95	(85.6%)	88	(73.3%)	0.024
IgM Antibody test	68	(29.4%)	26	(23.4%)	42	(35.0%)	0.061
RT-PCR	7	(3.0%)	2	(1.8%)	5	(4.2%)	0.295
Presenting symptoms							
Myalgia	204	(88.3%)	95	(85.6%)	109	(90.8%)	0.227
Headache	173	(74.9%)	86	(77.5%)	87	(72.5%)	0.448
Fatigue	143	(61.9%)	66	(59.5%)	77	(64.2%)	0.489
Retro-orbital pain	83	(35.9%)	41	(36.9%)	42	(35.0%)	0.785
Diarrhea	61	(26.4%)	24	(21.6%)	37	(30.8%)	0.136
Vomiting	57	(24.7%)	30	(27.0%)	27	(22.5%)	0.448
Abdominal discomfort	39	(16.9%)	21	(18.9%)	18	(15.0%)	0.484
Physical examinations							
Body temperature	38.0	(37.0-38.8)	38.1	(37.3-38.8)	38.0	(37.5-38.6)	0.714
Jaundice	4	(1.2%)	2	(1.8%)	2	(1.7%)	0.659
Hepatomegaly	144	(62.3%)	72	(64.9%)	72	(60.0%)	0.498
Petechiae	17	(7.4%)	6	(5.4%)	11	(9.2%)	0.320
Laboratory results at admission							
Hemoglobin (g/dL)	13.9	(12.5-15.8)	14.1	(13.0-15.2)	13.6	(12.5-14.7)	0.024
MCV (fL)	82.9	(75.0-87.3)	81.7	(76.3-87.3)	83.5	(75.6-86.6)	0.940
WBC (× 10⁹/L)	2.9	(2.2-5.2)	2.9	(2.3-4.5)	3.0	(2.4-4.3)	0.591
Neutrophil (%)	50.0	(32.0-62.0)	49.0	(36.0-62.0)	50.0	(38.0-62.0)	0.886
Lymphocyte (%)	28.0	(17.0-36.0)	29.0	(20.0−32.0)	27.5	(18.0-35.0)	0.556
Platelet count (× 10⁹/L)	73.0	(46.0-133.0)	71.0	(46.0-111.0)	74.0	(48.0-96.0)	0.762
AST (U/L)	125	(53-245)	128	(78-220)	123	(56-231)	0.413
ALT (U/L)	93	(43-188)	96	(46-188)	91	(47-184)	0.554
G6PD activity (U/gHb)	10.49	(7.36-11.16)	8.47	(7.41-10.07)	10.82	(10.45-11.08)	0.001
Treatment outcome							
Recovery	228	(98.7%)	109	(98.2%)	119	(99.2%)	0.609

Nonsteroidal Anti-Inflammatory Drugs (NSAIDs), Proton Pump Inhibitors (PPIs), Norethisterone (Primalut-N), White blood cells count (WBC), Aspartate Aminotransferase (AST), Alanine Aminotransferase (ALT).

Data are presented as median (IQR) or number (%).

**Fig 1:**
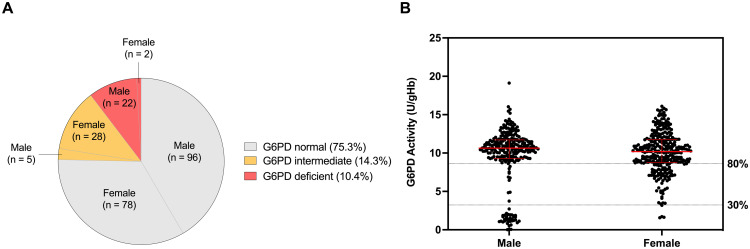
Prevalence of G6PD deficiency (A) and distribution of G6PD enzyme activity (B) in dengue samples. The AMM of the studied population was determined to be 10.76 ± 1.07 U/gHb. Based on the WHO classification, individuals with <30% activity (3.23 U/gHb), comprising 22 males and 2 females, were classified as G6PD deficient. Those with 30–80% activity (3.23–8.61 U/gHb), including 5 males and 28 females, were classified as G6PD intermediate. Individuals with >80% activity, consisting of 96 males and 78 females, were classified as G6PD normal.

G6PD activity was measured in all patients and the AMM was determined to be 10.76 ± 1.07 U/gHb [36]. Based on the World Health Organization criteria [44], individuals with <30% of normal activity were considered as G6PD deficient, those with 30‒80% of normal activity as G6PD intermediate, and those with >80% activity as G6PD normal. Accordingly, 10.4% (24/231) of patients were classified as G6PD deficiency, of whom 22 were males and 2 were females. Additionally, 14.3% (33/231) exhibited intermediate G6PD activity, with the majority being females (28/33) (Fig 1A). The distribution of G6PD activity in the studied population is shown in Fig 1B.

HRM analysis identified *G6PD* mutations in most of the patients with a deficient phenotype; however, three samples with a deficient phenotype had no detectable mutations by HRM. In addition, one female had borderline intermediate G6PD activity and was found to carry a combination of c.1311C > T and c.1365-13T > C mutations. These four samples underwent confirmatory DNA sequencing. Overall, *G6PD* mutations were identified in 111 patients (48.0%), encompassing 16 *G6PD* genotypes (Table 2).

**Table 2:** *G6PD* genotypes among dengue-positive samples.

Genotype	Variant name	n	Frequency
c.143T > C	Aures	1	0.9%
c.383T > C*	Vanua Lava	1	0.9%
c.392G > T	Chinese-4	4	3.6%
c.406C > T, c.1311C > T, c.1365-13T > C	Valladolid	1	0.9%
c.486−34delT		11	9.9%
c.486−34delT, c.1311C > T, c.1365-13T > C		2	1.8%
c.487G > A	Mahidol	2	1.8%
c.871G > A, c.1311C > T, c.1365-13T > C	Viangchan	20	18.0%
c.871G > A, c.1024C > T, c.1311C > T, c.1365-13T > C	Viangchan, Chinese-5,	1	0.9%
c.1311C > T, c.1365-13T > C		60	54.1%
c.1360C > T	Union	2	1.8%
c.1376G > T	Canton	2	1.8%
c.1376G > T, c.486−34delT	Canton,	1	0.9%
c.1388G > A*	Kaiping	2	1.8%
c.1439T > C*, c.1311C > T, c.1365-13T > C	Phaya Thai	1	0.9%
Total		111	100%

Bold indicates novel mutation identified in this study.

Asterisk indicates mutations identified by DNA sequencing.

The most common mutation was a combination of c.1311C > T (synonymous mutation) and c.1365-13T > C (intronic variant), accounting for 54.1% of mutation-positive individuals. This was followed by the compound mutation of *G6PD* Viangchan (c.871G > A, c.1311C > T, c.1365-13T > C; 18.0%), deletion variant (c.486−34delT; 9.9%) and *G6PD* Chinese-4 (c.392G > T; 3.6%). Additionally, five other *G6PD* genotypes with a lower frequency (1.8%) were identified, including compound mutations of deletion variant (c.486−34delT, c.1311C > T, c.1365-13T > C), *G6PD* Mahidol (c.487G > A), *G6PD* Union (c.1360C > T), *G6PD* Canton (c.1376G > T), and *G6PD* Kaiping (c.1388G > A). Interestingly, the double missense mutation *G6PD* Viangchan+Chinese-5 (c.871G > A, c. 1024C > T, c.1311C > T, c.1365-13T > C) and a new variant c.1439T > C were identified with a frequency of 0.9% each. The new variant was named “*G6PD* Phaya Thai”. Three other variants were also identified with a frequency of 0.9%, including *G6PD* Aures (c.143T > C), *G6PD* Vanua Lava (c.383T > C), and a combination of *G6PD* Canton along with deletion mutation (c.1376G > T, c.486−34delT).

Comparative analysis between individuals with (n = 111) and those without mutations (n = 120) revealed no significant difference in baseline demographics, self-medication practices, clinical symptoms, or most laboratory parameters. However, there were a few notable differences. NS1 antigen detection was significantly more frequent in patients with G6PD mutations (85.6%) compared to those without mutations (73.3%, p = 0.024). Additionally, patients carrying mutations had significantly higher median hemoglobin levels (14.1 g/dL) than those without mutations (13.6 g/dL, p = 0.024). G6PD enzyme activity was also significantly lower in the mutation group (median 8.47 U/gHb) compared to those without mutations (10.82 U/gHb, p = 0.001). No significant differences were observed in platelet count, liver enzyme levels, or treatment outcomes, with recovery achieved in over 98% of patients in both groups. There were three patient deaths (two in the *G6PD* mutation group and one in the non-mutation group) but this difference was not statistically significant. All deaths were attributed to multiorgan dysfunction; however, no autopsy data were available to confirm the underlying causes.

### G6PD phenotype-genotype association analysis

**Fig 2:**
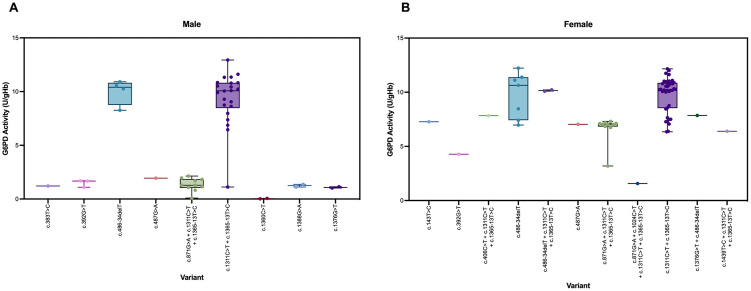
Box plot of G6PD activity for each variant among (A) male and (B) female samples.

The analysis of G6PD phenotype-genotype association in dengue samples is shown in Fig 2. All single missense mutations in hemizygotes and multiple missense mutations in compound heterozygotes showed deficient phenotypes. Single missense mutations in heterozygotes resulted in either intermediate or normal phenotype. A combination of c.1311C > T and c.1365-13T > C resulted in a wide range of enzyme activity, ranging from deficient to normal in males and intermediate to normal in females. An intronic variant c.1365-13T > C resulted in normal activity, while a deletion variant c.486−34delT showed intermediate and normal phenotypes.

Based on the classification system established in 1971 [45], out of 231 patients diagnosed with dengue infection, five males carrying *G6PD* mutations showed enzyme activity less than 10% (Table 3). Twenty-four individuals (18 males and 6 females) showed G6PD activity between 10–60%, all of whom carried *G6PD* mutations. Furthermore, 202 individuals (100 males and 102 females) exhibited G6PD activity greater than 60%, with 82 of them (24 males and 58 females) carrying *G6PD* mutations. No *G6PD* mutations were detected in 120 individuals and all had G6PD activity greater than 70%. Therefore, they were classified as samples without mutations. However, they may carry mutations in intronic regions, which do not affect enzyme activity.

**Table 3:** *G6PD* genotyping and enzyme activity among 231 adult patients with dengue infection.

G6PD activity	Sex	n (%)	Samples with mutations n (%)	Samples without mutations n (%)
60‒150%	Male	100 (49.5)	24 (29.3)	76 (63.3)
	Female	102 (50.5)	58 (70.7)	44 (36.7)
10‒60%	Male	18 (75.0)	18 (75.0)	0
	Female	6 (25.0)	6 (25.0)	0
<10%	Male	5 (100)	5 (100)	0
	Female	0	0	0
**Total**		**231**	**111 (48.1)**	**120 (51.9)**

### Association of G6PD deficiency and hemoglobin levels in dengue patients

**Fig 3:**
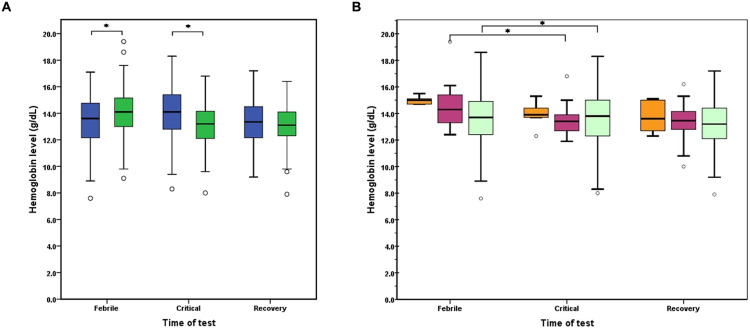
The variation in hemoglobin levels over the course of illness among adult dengue patients. (A) The hemoglobin level changes in patients without mutations (blue box) and those carrying *G6PD* mutations (dark green box). (B) The hemoglobin level changes during the illness timeline in patients carrying *G6PD* mutations with different enzyme activity levels: < 10% (orange box), between 10‒60% (purple box), and 60‒150% (light green box), respectively. Asterisk indicates statistical significance (*p*-value < 0.05).

Among the 111 individuals (48.1%) carrying *G6PD* mutations, it was found that these mutations were associated with changes in hemoglobin levels during the course of dengue illness compared to those without mutations. During the critical phase, adult dengue patients with mutations showed a significant drop in hemoglobin levels, compared to the febrile phase. Conversely, patients without mutations had an increase in hemoglobin levels during the critical phase (Fig 3A). There was a significant difference in median hemoglobin levels between patients with and without mutations during the febrile (14.1; IQR: 13.0–15.2 g/dL *vs* 13.6; IQR: 12.1–14.8 g/dL; *p *= 0.02), and the critical phase (13.2; IQR: 12.1–14.2 g/dL *vs* 14.1; IQR: 12.7–15.4 g/dL; *p *< 0.001). However, no significant difference was observed in median hemoglobin levels between two groups during recovery phase (13.1; IQR: 12.3–14.1 g/dL *vs* 13.3; IQR: 12.1–14.5 g/dL; *p *= 0.24). In contrast, in the group without mutations, hemoglobin levels increased (*p* = 0.013) from the febrile to the critical phase, followed by a decrease (*p* = 0.498) during the recovery phase. In individuals with *G6PD* mutations, each subgroup categorized by three activity levels demonstrated a decline in hemoglobin levels across all phases (Fig 3B). There was a significant difference of hemoglobin levels between the febrile and the critical phase among patients with G6PD activity between 10‒60% and >60% enzyme activity (*p *= 0.002 and 0.013, respectively).

It should be noted that all patients received fluid management tailored to their clinical status, in accordance with dengue management guidelines. This treatment aimed to maintain effective circulation and prevent shock but did not directly affect hemoglobin levels, aside from the expected hemoconcentration and dilution effects inherent to dengue pathophysiology. Additionally, the use of NSAIDs and other drugs that might influence bleeding or hemoglobin levels was prohibited during the illness. No other pharmacologic interventions known to affect hemoglobin concentrations were administered during the febrile or critical phases.

The observed variability in hemoglobin levels among the three groups of enzyme activity across illness phases may not be fully explained by clinical management alone. While bleeding could contribute to changes in hemoglobin, the clinical data did not indicate significant bleeding events among these patients. Therefore, hemolysis is the most likely explanation for the differences observed during the febrile phase, particularly in patients with G6PD deficiency.

This is consistent with evidence that G6PD-deficient individuals may experience hemolysis triggered by viral infections and febrile episodes [25,46]. Such hemolysis can counteract the typical hemoconcentration seen in dengue, which explains why patients with G6PD deficiency did not exhibit the marked hemoconcentration observed in non–G6PD-deficient groups. However, severe hemolysis was not observed, likely due to the specific G6PD mutations identified in this study and their associated functional severity profiles.

Considering hemoglobin levels in association with *G6PD* variants (Table 4), a decrease in hemoglobin levels during the critical phase was observed across all *G6PD* variants. Seven *G6PD* variants‒c.1311C > T, c.1365-13T > C; c.871G > A, c.1311C > T, c.1365-13T > C; c.486−34delT; c.392G > T; c.487G > A; c.1376G > T; c.1388G > A‒demonstrated persistently low hemoglobin levels during recovery phase. When comparing hemoglobin levels between individuals without mutations and those with specific *G6PD* variants, statistically significant differences were observed during the dengue critical phase for two *G6PD* variants: a combination of c.1311C > T and c.1365-13T > C and the compound mutation of *G6PD* Viangchan (*p* = 0.02 and 0.01, respectively). With a sample size of 231, certain *G6PD* variants (Aures, Vanua Lava, Valladolid, Viangchan + Chinese-5, Canton, and Phaya Thai) were excluded from the individual *G6PD* variant analysis due to their low frequency (one patient each). Similarly, the triple mutation (c.486−34delT, c.1311C > T, c.1365-13T > C), as well as the Mahidol, Union, Canton, and Kaiping variants, were excluded, as they were present in only two patients each.

**Table 4:** Changes in hemoglobin levels during dengue phases, stratified by *G6PD* variant.

Genotype	Variant name	n	Hemoglobin levels across dengue phase^*^		
			Febrile	Critical	Recovery
c.1311C > T, c.1365-13T > C		60	14.0 (13.0-14.9)	13.1^**^ (12.0-14.2)	13.2 (12.4-14.2)
c.871G > A, c.1311C > T, c.1365-13T > C	Viangchan	20	14.2 (12.8-15.1)	12.5^**^ (12.0-13.7)	13.1 (11.9-13.9)
c.486−34delT		11	13.3 (12.7-15.1)	13.0 (12.0-14.7)	12.9 (11.3-14.2)
c.392G > T	Chinese-4	4	14.7 (14.5-15.2)	14.0 (13.6-15.5)	13.9 (13.4-14.8)

*Median (IQR) (g/dL).

**Hemoglobin levels were compared between individuals with and without the corresponding *G6PD* genotype (*p* < 0.05).

### Biochemical and structural characterization of G6PD variants

Understanding how specific mutations lead to enzyme deficiency is essential. At the protein level, reduced catalytic activity and/or structural instability contribute to the deficient phenotype. Steady-state kinetics were determined to evaluate catalytic activity and substrate affinity of G6PD variants (Table 5). G6PD Viangchan and G6PD Chinese-5 exhibited reduced catalytic activity but showed greater binding affinity toward G6P substrate compared to the WT enzyme. The novel variant, G6PD Phaya Thai, demonstrated slightly increased catalytic activity but lower binding affinity for both substrates. The double mutant (Viangchan+Chinese-5) displayed the most severe reduction in enzyme activity, though substrate binding affinity remained comparable to WT.

**Table 5:** Kinetic parameters of recombinant G6PD variants.

Construct	Amino acid change	k_cat_ (s^–1^)	K_m_ G6P (μM)	K_m_ NADP^+^ (μM)
WT	–	354.3 ± 7.5	42.1 ± 3.6	8.3 ± 2.2
Phaya Thai	Ile480Thr	392.3 ± 7.4	60.9 ± 3.8	27.6 ± 7.7
Viangchan	Val291Met	121.6 ± 2.0	34.6 ± 2.0	14.0 ± 4.3
Chinese-5	Leu342Phe	183.8 ± 19.2	36.9 ± 7.8	9.1 ± 3.9
Viangchan+Chinese-5	Val291Met+Leu342Phe	157.8 ± 12.8	39.2 ± 4.2	9.6 ± 3.5

CD spectroscopy showed no major disruption to the secondary structure in any variant, with all proteins maintaining characteristic α-helical features (S1 Figure in S2 File). However, subtle changes in flexibility or rigidity were observed in G6PD Phaya Thai and G6PD Viangchan+Chinese-5.

**Fig 4:**
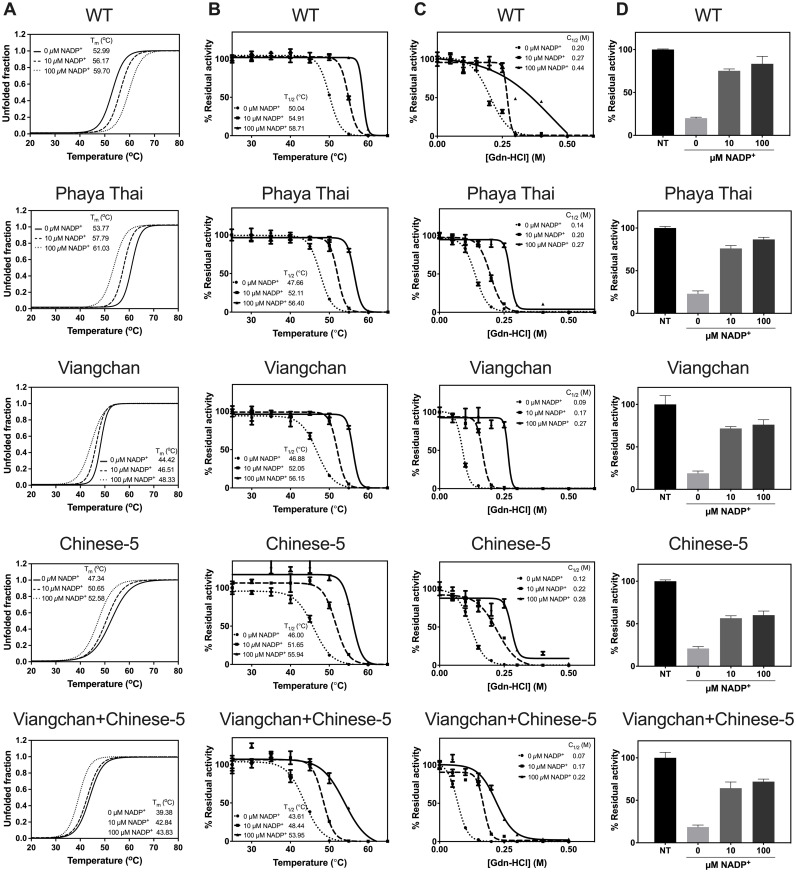
Structural stability analyses of G6PD variants. (A) Thermal stability analysis, with *T*_*m*_ defined as the temperature at which half of the protein unfolded. (B) Thermal inactivation analysis, *T*_*1/2*_ was defined as the temperature at which the enzyme loses 50% of its activity. (C) Susceptibility to Gdn-HCl treatment, with *C*_*1/2*_ defined as the concentration of Gdn-HCl at which the enzyme loses 50% of its activity. (D) Susceptibility to trypsin digestion. Residual enzyme activity was measured after trypsin treatment and expressed as a percentage of the activity of the same enzyme incubated without trypsin.

G6PD structural stability is also a key determinant of clinical phenotype. Thermal shift assay revealed that single missense G6PD mutations altered structural stability (Fig 4A and S2 Table in S1 File), with *T*_*m*_ of 44.42°C for G6PD Viangchan and 47.34°C for G6PD Chinese-5, compared to 52.99°C for G6PD WT. The double mutant, G6PD Viangchan+Chinese-5, was the least stable enzyme (*T*_*m *_= 39.38°C). In contrast, G6PD Phaya Thai, had a *T*_*m*_ of 53.77 °C, similar to WT. The presence of structural NADP^+^ increased *T*_*m*_ across all variants, confirming its stabilizing role.

Thermal inactivation studies further supported these findings (Fig 4B and S3 Table in S1 File). G6PD Phaya Thai had *T*_*1/2*_ of 47.66 °C, while G6PD Viangchan+Chinese-5 showed the lowest stability (*T*_*1/2* _= 43.61 °C). WT enzyme exhibited a *T*_*1/2*_ of 50.04 °C.

As shown in Fig 4C and S4 Table in S1 File, chemical denaturation using Gdn-HCl confirmed that all G6PD variants are structurally less stable than the G6PD WT (*C*_*1/2*_ = 0.20 M). The double mutant had the lowest resistance (*C*_*1/2*_ = 0.07 M), followed by G6PD Viangchan (*C*_*1/2*_ = 0.09), G6PD Chinese-5 (*C*_*1/2*_ = 0.12 M), and G6PD Phaya Thai (*C*_*1/2*_ = 0.14 M), respectively.

Finally, trypsin digestion assays showed similar trends (Fig 4D and S5 Table in S1 File). In the absence of NADP^+^, all variants retained enzyme activity levels comparable to that of the WT enzyme (20.06%). G6PD Phaya Thai had the highest residual activity (22.99%), while the double mutant retained the least (18.54%). These results consistently demonstrate that the G6PD Viangchan+Chinese-5 variant resulted in the least structurally stable protein among G6PD variants studied here.

### Molecular docking and molecular dynamic simulations

Molecular dynamic simulation was utilized to investigate how G6PD mutations affect protein structure, multimerization and binding to G6P substrate and NADP^+^ cofactors. The G6PD Viangchan+Chinese-5 double variant showed structural changes similar to its corresponding single variants (S6 Table in S1 File). In G6PD Chinese-5 variant, the Lue342Phe substitution, while not affecting hydrogen bonds between βI and βJ, caused local shifts due to the bulkier side chain. The Val291Met mutation in G6PD Viangchan led to increased exposure of neighboring residues, increasing the αj and the αj – αk loop distance from 1.6 Å to 6.8 Å. The novel G6PD Phaya Thai variant (Ile480Thr) caused minimal structural changes, reducing the distance between residue 480 and residues 305–307 from 10.4 Å to 9.7 Å.

RMSF analysis (S7 Table in S1 File) showed greater flexibility at the active and multimerization sites in G6PD Chinese-5 and G6PD Viangchan+Chinese-5, correlating with their lower catalytic activity. The G6PD Chinese-5 variant maintained hydrogen bonding between Lys-171 and G6P substrate and NADP^+^ cofactors, whereas G6PD Viangchan+Chinese-5 and G6PD Viangchan lost these key interactions during the simulation, explaining their reduced activity (S2 Figure in S2 File).

**Fig 5:**
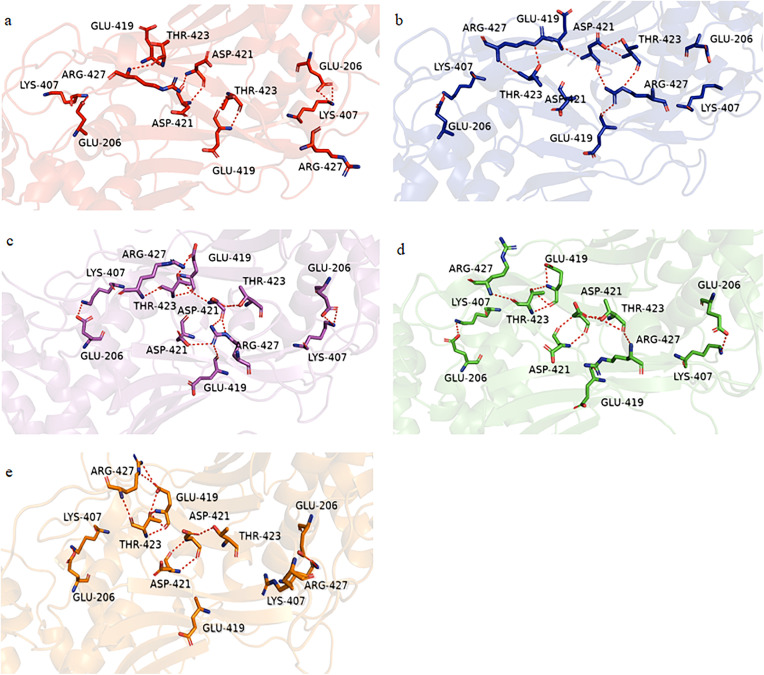
Trajectory snapshots of the (A) G6PD WT (red), (B) G6PD Phaya Thai (blue), (C) G6PD Chinese-5 (magenta), (D) G6PD Viangchan (green) and (E) G6PD Viangchan+Chinese-5 (orange) at the dimer interface.

Despite mutations at the tetramer interface, the G6PD Viangchan+Chinese-5 variant preserved hydrogen bonds and salt bridges at the dimer interface (Fig 5, S8 Table in S1 File). However, SASA analysis indicated impaired tetramerization (SASA = 19.32 nm^2^ vs. 20.50 nm^2^ in WT), potentially reducing enzymatic activity. This variant also showed a higher Rg, supporting a more unstable structure. In contrast, G6PD Viangchan’s activity loss was mainly due to high RMSF at active site and multimer interfaces (S7 Table in S1 File).

In G6PD Phaya Thai, the dimer and tetramer remained stable (S8 Table in S1 File), with only a slight shift in βN-βN sheets (2.21 Å vs. 2.10 Å in WT). Minor conformational changes in the βe-αe loop led to fewer hydrogen bonds with G6P and NADP^+^, consistent with the modest kinetic changes (S2 Figure in S2 File). Overall, G6PD Phaya Thai remains structurally similar to the WT enzyme.

### Discussion

This study presents a comprehensive assessment of G6PD deficiency among adult dengue patients in Thailand, offering new insights into both its prevalence and molecular spectrum of *G6PD* variants. Uniquely, it is the first to simultaneously evaluate G6PD enzyme activity and genotype in this population, providing a more complete understanding of genotype-phenotype relationships in the context of acute viral infection.

The observed prevalence of G6PD deficiency was 10.4%, consistent with previous reports in Thailand [37,47–49]. Importantly, a wide and previously underrecognized diversity of *G6PD* variants was identified. This genetic heterogeneity reflects the evolutionary pressure, particularly from malaria, that has shaped the regional distribution of G6PD variants in Southeast Asia [50,51]. In addition to common variants such as G6PD Viangchan, G6PD Mahidol and G6PD Canton, two novel variants‒G6PD Viangchan+Chinese-5 and G6PD Phaya Thai‒were identified. These observations highlight the need for comprehensive molecular surveillance in genetically diverse populations.

As expected, male patients carrying missense mutations consistently exhibited deficiency, while female carriers showed either deficient or intermediate deficiency phenotypes due to random X-chromosome inactivation [52]. Functional characterization confirmed that most variants impaired enzyme activity due to impaired structural stability [53–57]. Notably, the compound Viangchan+Chinese-5 mutation resulted in a more severe enzyme deficiency than its corresponding single mutations, suggesting additive or synergistic effects on protein function. In contrast, the novel G6PD Phaya Thai variant had only a minor effect on structural stability, with preserved dimer and tetramer interfaces and showed catalytic activity comparable to the WT enzyme. This highlights the importance of integrating enzymatic assays with molecular diagnostics to detect clinically relevant variants that may otherwise be overlooked.

Although traditionally considered benign, mutations such as c.1311C > T and c.1365-13T > C were detected in patients with varying enzyme activity levels. For instance, one male carrying both mutations exhibited severe deficiency (1.12 U/gHb), whereas others with the same genotype showed normal or intermediate phenotype. These mutations, while common in general population [37,58,59], have been found to associate with enzyme deficiency in certain populations [60,61]. This variability suggests potential roles in post-transcriptional regulation, possibly affecting mRNA stability, splicing or translational efficiency [62]. To determine their clinical significance, future functional studies, such as transcriptome analysis, splicing assays, or *in vitro* expression models, are essential.

Importantly, the study provides a disease-relevant context by analyzing *G6PD* variants in patients with dengue infection, a condition known to induce oxidative stress. During dengue infection, the inflammatory response and immune activation contribute to elevated reactive oxygen species (ROS), particularly during the febrile and early critical phases [63]. G6PD-deficient individuals, with reduced ability to detoxify ROS due to impaired NADPH production, are particularly vulnerable to oxidative stress-induced hemolysis.

Patients with *G6PD* mutations showed a decline in hemoglobin levels from the febrile to the critical phase, whereas those without mutations experienced an increase, likely due to plasma leakage and hemoconcentration [28]. This pattern supports the hypothesis that oxidative stress in dengue may trigger subclinical or overt hemolysis in G6PD-deficient patients, consistent with previous findings in children [25]. However, due to the lack of hemolysis markers (e.g., haptoglobin, lactate dehydrogenase, bilirubin), this proposed mechanism remains speculative and highlights the need for future prospective studies.

Subgroup analysis based on G6PD enzyme levels revealed that even individuals with intermediate and high G6PD activity experienced significant hemoglobin declines, suggesting that partial deficiency may also increase susceptibility to oxidative stress. Interestingly, individuals with severe G6PD deficiency (<10% activity) did not show marked changes, possibly due to the small sample size‒only 2.2% of the total studied population‒limiting statistical power and generalizability. Larger studies are needed to further explore the impact of rare and severe *G6PD* variants.

Notably, significant hemoglobin declines during the critical phase were observed in individuals carrying both the c.1311C > T and c.1365-13T > C mutations, as well as those with the compound mutation of *G6PD* Viangchan. These findings correlate with biochemical and structural evidence of protein instability and highlight the importance of monitoring hemoglobin levels in G6PD-deficient patients during dengue infection.

These distinct hemoglobin trends raise potential therapeutic considerations. Transfusion protocols may need to be tailored for dengue patients with G6PD deficiency, especially those showing early signs of hemolysis. Although current evidence is limited to case reports and series [27,64], tailored interventions may help prevent complications related to anemia and tissue hypoxia in this vulnerable population.

Several limitations must be acknowledged. The retrospective nature of the study and the lack of information on disease severity, dengue serotype, and transfusion practices constrain causal inference [65,66]. Additionally, other hemolytic conditions, such as hemoglobinopathies, were not explored, despite their potential to influence hemoglobin dynamics [67,68]. Future prospective studies incorporating broader clinical and laboratory data are needed to address these gaps.

In summary, this study provides detailed molecular and functional characterization of *G6PD* variants in Thai dengue patients and sheds light on the interaction between host genetic factors and infection-induced oxidative stress. The observed hemoglobin fluctuations suggest that G6PD deficiency may influence disease course in dengue and should be considered in clinical care. These findings support integrating G6PD status into dengue management protocols, particularly in regions with high prevalence of G6PD deficiency.

## Supporting information

S1 File **Table File: S1 Table.** Primers used for site-directed mutagenesis. **S2 Table.** Melting temperature (T_m_) values of recombinant G6PD proteins by thermal shift assay. **S3 Table.** Thermal inactivation of G6PD variants. **S4 Table.** Stability of G6PD variants in the presence of Gdn-HCl. **S5 Table.** Susceptibility of G6PD variants to trypsin digestion. **S6 Table.** Distance between the mutation site and neighboring residues for the WT and G6PD variants. **S7 Table.** Average values of the trajectory analyses performed on the WT and variants. **S8 Table.** Structural characteristics of the dimer and tetramer interface (t = 100 ns).
(DOCX)

S2 File **Figure File: S1 Figure.** Secondary structure analysis of G6PD variants by circular dichroism (CD) spectroscopy. spectroscopy. **S2 Figure.** Ligand binding pocket occupancy heatmap indicating the presence (red) and absence (blue) of hydrogen bonds (t = 100 ns).
(DOCX)

S3 Raw data Raw data file. (XLSX)
