# Supplementary material for: Spectrum of glucose-6-phosphate dehydrogenase (G6PD) mutations and trends in hemoglobin levels among adult dengue patients in Thailand
Source: PLoS One. 2025 Sep 18;20(9):e0332039. doi: 10.1371/journal.pone.0332039 (PMC12445492; doi:10.1371/journal.pone.0332039)
Supplement: S1 File — Table File: S1 Table. Primers used for site-directed mutagenesis. S2 Table. Melting temperature (Tm) values of recombinant G6PD proteins by thermal shift assay. S3 Table. Thermal inactivation of G6PD variants. S4 Table. Stability of G6PD variants in the presence of Gdn-HCl. S5 Table. Susceptibility of G6PD variants to trypsin digestion. S6 Table. Distance between the mutation site and neighboring residues for the WT and G6PD variants. S7 Table. Average values of the trajectory analyses performed on the WT and variants. S8 Table. Structural characteristics of the dimer and tetramer interface (t = 100 ns). (DOCX) [file pone.0332039.s001.docx]

**Supporting information-Table**

**Spectrum of glucose-6-phosphate dehydrogenase (G6PD) mutations and trends in hemoglobin levels among adult dengue patients in Thailand**

Supat Chamnanchanunt^1^, Beatriz Aira C Jacob^2^, Vipa Thanachartwet^1^, Varunee Desakorn^1^, Natsamon Singha-art^2^, Duangjai Sahassananda^3^, Kamonwan Chamchoy^4^, Naveen Eugene Louis^5^, Muawiaa Ahmed Hamza^6^, Nurriza Ab Latif^5^, Syazwani Itri binti Amran^5^, Henry A F Stephens^7^, Wang Nguitragool^2^, Usa Boonyuen^2,*^

^1^ Department of Clinical Tropical Medicine, Faculty of Tropical Medicine, Mahidol University, Bangkok, Thailand

^2^ Department of Molecular Tropical Medicine and Genetics, Faculty of Tropical Medicine, Mahidol University, Bangkok, Thailand

^3^ Information Technology Unit, Faculty of Tropical Medicine, Mahidol University, Bangkok, Thailand

^4^ Princess Srisavangavadhana Faculty of Medicine, Chulabhorn Royal Academy, Bangkok, Thailand

^5^ Department of Bioscience, Faculty of Science, Universiti Teknologi Malaysia (UTM), Johor Bahru, Malaysia

^6^ Faculty of Medicine, King Fahad Medical City, Riyadh, Saudi Arabia

^7^ Department of Renal Medicine, University College London (UCL), Royal Free Hospital, Rowland Hill Street, London, United Kingdom

**Corresponding author**: Usa Boonyuen

Email: usa.boo@mahidol.ac.th

**S1 Table.** Primers used for site-directed mutagenesis.

| **Primer** | **Sequence (5’ to 3’)** |
| --- | --- |
| Viangchan_F | GATGAGAAGGTCAAGATGTTGAAATGCATC |
| Viangchan_R | GATGCATTTCAACATCTTGACCTTCTCATC |
| Chinese-5_F | CACTTTTGCAGCCGTCGTCT |
| Chinese-5_R | AGACGACGGCTGCAAAAGTG |
| Phaya Thai_F | CCCAAGCCCACCCCCTATAT |
| Phaya Thai_R | ATATAGGGGGTGGGCTTGGG |

**S2 Table.** Melting temperature (*T_m_*) values of recombinant G6PD proteins by thermal shift assay.

| **Construct** | ***T_m_* (°C)** | | |
| --- | --- | --- | --- |
|  | **0 µM NADP^+^** | **10 µM NADP^+^** | **100 µM NADP^+^** |
| WT  Phaya Thai | 52.99  53.77 | 56.17  57.79 | 59.70  61.03 |
| Viangchan | 44.42 | 46.51 | 48.33 |
| Chinese-5 | 47.34 | 50.65 | 52.58 |
| Viangchan+Chinese-5 | 39.38 | 42.84 | 43.83 |

**S3 Table.** Thermal inactivation of G6PD variants.

| **Variant** | ***T_1/2_* (°C)** | | |
| --- | --- | --- | --- |
|  | **0 μM NADP^+^** | **10 μM NADP^+^** | **100 μM NADP^+^** |
| WT  Phaya Thai | 50.04  47.66 | 54.91  52.11 | 58.71  56.40 |
| Viangchan | 46.88 | 52.05 | 56.15 |
| Chinese-5 | 46.00 | 51.65 | 55.94 |
| Viangchan+Chinese-5 | 43.61 | 48.44 | 53.95 |

**S4 Table.** Stability of G6PD variants in the presence of Gdn-HCl.

| **Variant** | ***C_1/2_* (M)** | | |
| --- | --- | --- | --- |
|  | **0 μM NADP^+^** | **10 μM NADP^+^** | **100 μM NADP^+^** |
| WT  Phaya Thai | 0.20  0.14 | 0.27  0.20 | 0.44  0.27 |
| Viangchan | 0.09 | 0.17 | 0.27 |
| Chinese-5 | 0.12 | 0.22 | 0.28 |
| Viangchan+Chinese-5 | 0.07 | 0.17 | 0.22 |

**S5 Table.** Susceptibility of G6PD variants to trypsin digestion.

| **Variant** | **% Residual activity** | | | |
| --- | --- | --- | --- | --- |
|  | **NT** | **0 μM NADP^+^** | **10 μM NADP^+^** | **100 μM NADP^+^** |
| WT  Phaya Thai | 100±0.68  100±1.89 | 20.06±1.18  22.99±3.29 | 75.24±2.15  76.09±3.39 | 83.39±8.63  86.62±2.50 |
| Viangchan | 100±10.33 | 18.95±2.62 | 71.69±2.20 | 76.13±5.83 |
| Chinese-5 | 100±1.56 | 20.89±2.20 | 56.50±2.82 | 60.13±4.80 |
| Viangchan+Chinese-5 | 100±6.40 | 18.54±2.33 | 64.45±7.27 | 71.96±2.99 |

**S6 Table**. Distance between the mutation site and neighboring residues for the WT and G6PD variants.

| **Mutation site** | | | **Distance (Å)** | | | |
| --- | --- | --- | --- | --- | --- | --- |
| **Variant** | **Position** | **Mutated residue** | **Neighboring residue** | **WT** | **Variant** |  |
| Phaya Thai | 480 | Ile ->Thr  (βO–αo loop 474 - 489) | Leu 305 & Gln 307  (βH 304–310) | 10.4 | 9.7 |  |
| Viangchan | 291 | Val->Met  (αj 281-292) | Cys 294 (αj - αk loop 293-299) | 1.6 | 6.8 |  |
| Chinese-5 | 342 | Leu->Phe  (βI 337–342) | Phe 354 (βJ 353 – 360) | 7.5 | 4.9 |  |
| Viangchan+Chinese-5 | 291 | Val->Met  (αj 281-292) | Cys 294 (αj - αk loop 293-299) | 1.6 | 6.8 |  |
|  | 342 | Leu->Phe  (βI 337–342) | Phe 354 (βJ 353 – 360) | 7.5 | 4.9 |  |

**S7 Table**. Average values of the trajectory analyses performed on the WT and variants.

|  | Molecular dynamic parameters | | | | | | | | |
| --- | --- | --- | --- | --- | --- | --- | --- | --- | --- |
| G6PD | RMSD | Rg | Hbond | SASA | RMSF | | | | |
|  |  |  |  |  | c.NADP^+^-binding site | G6P binding site | s.NADP^+^-binding site | Dimer Interface | Tetramer Interface |
| WT | 0.40 | 3.63 | 774.48 | 20.50 | 0.17 | 0.17 | 0.21 | 0.21 | 0.22 |
| Phaya Thai | 0.35 | 3.69 | 789.41 | 20.54 | 0.17 | 0.15 | 0.18 | 0.20 | 0.19 |
| Viangchan | 0.33 | 3.67 | 782.89 | 20.69 | 0.25 | 0.22 | 0.29 | 0.29 | 0.34 |
| Chinese-5 | 0.37 | 3.68 | 785.89 | 20.53 | 0.16 | 0.11 | 0.15 | 0.17 | 0.15 |
| Viangchan+Chinese-5 | 0.33 | 3.71 | 792.80 | 19.32 | 0.15 | 0.11 | 0.14 | 0.16 | 0.14 |

**S8 Table**. Structural characteristics of the dimer and tetramer interface (t = 100 ns).

| Dimer | | | | | | Tetramer | |
| --- | --- | --- | --- | --- | --- | --- | --- |
| Protein | βN - βN H-bonds | | βN - βN distance (Å) | Salt bridges at the dimer interface | | SASA of tetramer salt bridge residues (nm^2^) |  |
|  | Asp 421 - Asp 421 | Glu 419 - Thr 423 |  | Glu 206 - Lys 407 | Glu 419 - Arg 427 |  |  |
| WT | + | + | 2.10 | + | - | 20.50 |  |
| Phaya Thai | - | + | 2.21 | - | + | 20.54 |  |
| Viangchan | + | + | 2.15 | + | - | 20.69 |  |
| Chinese-5 | - | + | 2.16 | + | + | 20.53 |  |
| Viangchan+Chinese-5 | + | + | 2.17 | + | + | 19.32 |  |
