# Supplementary material for: Spectrum of glucose-6-phosphate dehydrogenase (G6PD) mutations and trends in hemoglobin levels among adult dengue patients in Thailand
Source: PLoS One. 2025 Sep 18;20(9):e0332039. doi: 10.1371/journal.pone.0332039 (PMC12445492; doi:10.1371/journal.pone.0332039)
Supplement: S2 File — Figure File: S1 Figure. Secondary structure analysis of G6PD variants by circular dichroism (CD) spectroscopy. spectroscopy. S2 Figure. Ligand binding pocket occupancy heatmap indicating the presence (red) and absence (blue) of hydrogen bonds (t = 100 ns). (DOCX) [file pone.0332039.s002.docx]

**Supporting information-Figure**

**Spectrum of glucose-6-phosphate dehydrogenase (G6PD) mutations and trends in hemoglobin levels among adult dengue patients in Thailand**

Supat Chamnanchanunt^1^, Beatriz Aira C Jacob^2^, Vipa Thanachartwet^1^, Varunee Desakorn^1^, Natsamon Singha-art^2^, Duangjai Sahassananda^3^, Kamonwan Chamchoy^4^, Naveen Eugene Louis^5^, Muawiaa Ahmed Hamza^6^, Nurriza Ab Latif^5^, Syazwani Itri binti Amran^5^, Henry A F Stephens^7^, Wang Nguitragool^2^, Usa Boonyuen^2,*^

^1^ Department of Clinical Tropical Medicine, Faculty of Tropical Medicine, Mahidol University, Bangkok, Thailand

^2^ Department of Molecular Tropical Medicine and Genetics, Faculty of Tropical Medicine, Mahidol University, Bangkok, Thailand

^3^ Information Technology Unit, Faculty of Tropical Medicine, Mahidol University, Bangkok, Thailand

^4^ Princess Srisavangavadhana Faculty of Medicine, Chulabhorn Royal Academy, Bangkok, Thailand

^5^ Department of Biosciences, Faculty of Science, Universiti Teknologi Malaysia (UTM), Johor Bahru, Malaysia

^6^ Faculty of Medicine, King Fahad Medical City, Riyadh, Saudi Arabia

^7^ Department of Renal Medicine, University College London (UCL), Royal Free Hospital, Rowland Hill Street, London, United Kingdom

**Corresponding author**: Usa Boonyuen

Email: usa.boo@mahidol.ac.th


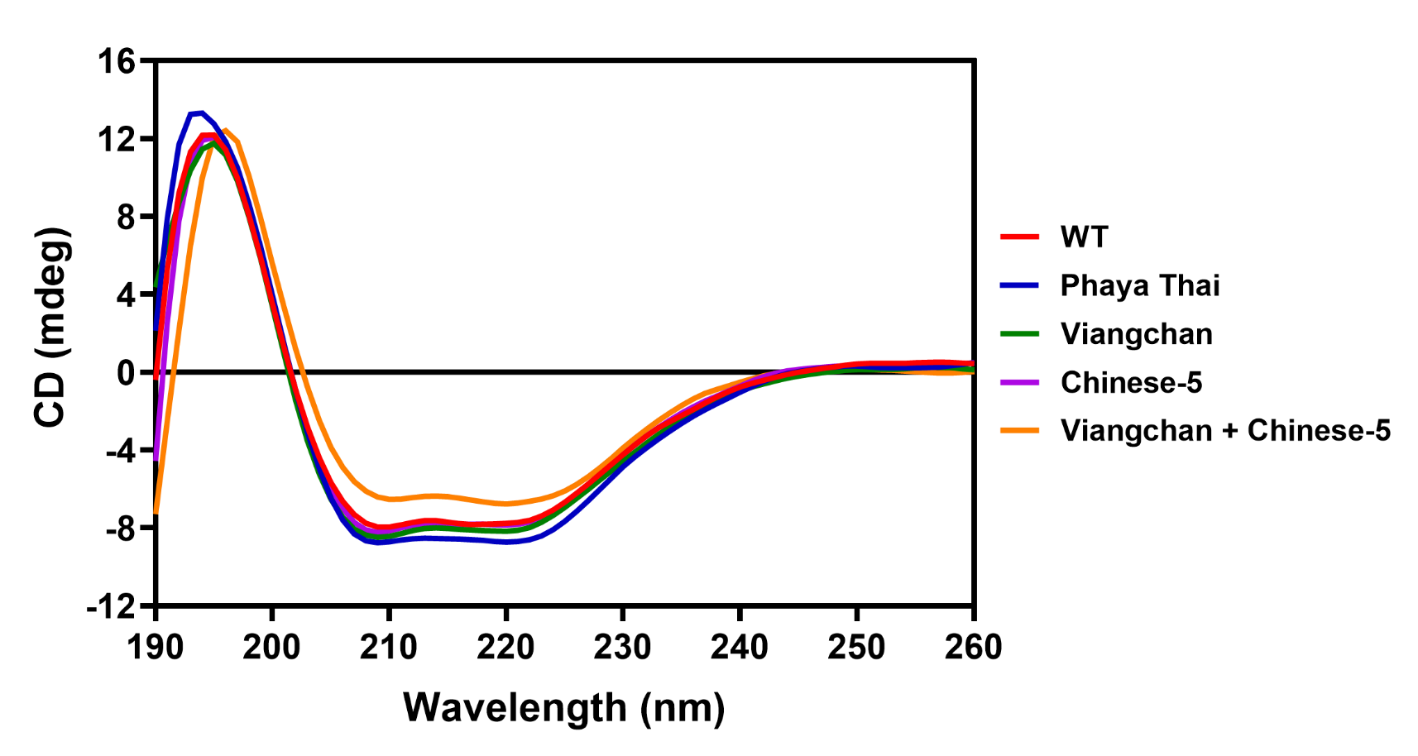


**S1 Figure**. Secondary structure analysis of G6PD variants by circular dichroism (CD) spectroscopy.


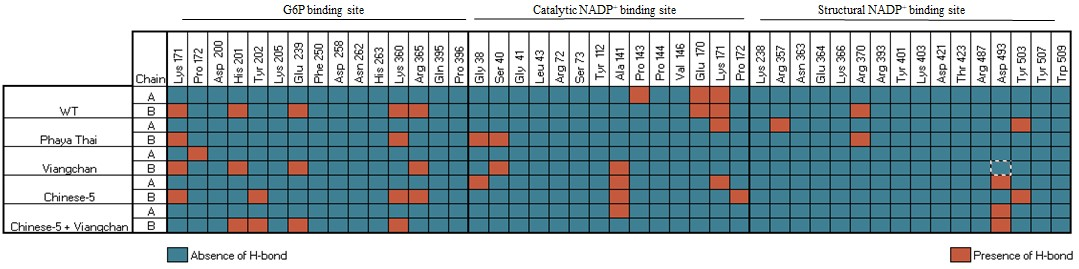


**S2 Figure**. Ligand binding pocket occupancy heatmap indicating the presence (red) and absence (blue) of hydrogen bonds (t = 100 ns).
